# Outcomes of endovascular treatment for patients with TASC II D femoropopliteal occlusive disease: a single center study

**DOI:** 10.1186/s12872-015-0025-1

**Published:** 2015-05-29

**Authors:** Xiangjiang Guo, Guanhua Xue, Xiaozhong Huang, Hui Xie, Wei Liang, Jiwei Zhang, Feng Lin, Tianping Yao

**Affiliations:** Department of Vascular Surgery, Renji Hospital, School of Medicine, Shanghai Jiaotong University, Pujian Road 160, Shanghai, 200127 China; Shanghai Testing & Inspection center for Medical Devices, Shanghai, China

**Keywords:** Peripheral artery disease, TASC II D lesions, Endovascular treatment, Patency rate, Risk factor

## Abstract

**Background:**

Advances in endovascular technology led to an alternative treatment option for TASC II D (TransAtlantic Inter-Society Consensus II class D) lesions. This study was aimed to evaluate the outcomes of endovascular treatment for TASC II D femoropopliteal lesions.

**Methods:**

Endovascular intervention with bare nitinol stent implantation was performed on 58 limbs (53 patients) with TASC II D femoropopliteal lesions from January 2011 to March 2013. Kaplan-Meier curves of primary patency, assisted patency and second patency were performed. Predictive factors of re-stenosis/occlusion were evaluated by univariate methods.

**Results:**

Total 53 patients with mean age of 74.2 ± 8.2 (range, 58.0–91.0 years) and mean lesion length of 314.8 ± 64.3 mm (188.2–400.4 mm) were enrolled. The mean follow-up time was 12.2 ± 6.1 months (5–38 months). Revascularization was successfully on 95 % lesions by bare nitinol stent implantation. Primary patency rates at 1, 2 and 3 years were 63 %, 12 % and 12 %, respectively. Assisted primary patency rates at 1, 2 and 3 years were 77 %, 31 % and 31 %, respectively. Secondary patency rates at 1, 2 and 3 years were 96 %, 63 % and 63 %. During one-year follow-up, no major amputation was occurred. Univariate analysis revealed that number of run-off vessels was a potential predictor of re-stenosis/occlusion.

**Conclusion:**

Endovascular treatment of TASC II D femoropopliteal artery occlusion has a high technical success rate with acceptable one-year patency rate. The long-term outcomes are poor, but endovascular intervention could be a good alternative for patients unsuitable for surgical bypass.

## Background

Peripheral artery disease (PAD) is a common circulatory problem, which refers to the obstruction of blood flow in the arteries exclusive of the coronary and cerebral vessels. Patients with PAD may suffer from claudication, ischemic rest pain, ischemic ulcerations and limb loss which consequently results in a poor quality of life and a high rate of depression. Treatment of PAD comprises conservative management for symptoms of claudication, such as risk factor modification, exercise therapy and antithrombotic therapy, and catheter-based or surgical revascularization for patients with critical limb ischemia (CLI) [1–3]. Endovascular treatment is an attractive alternative to open surgical procedures for PAD due to the less procedural risk resulted by it. The recent TransAtlantic InterSociety Consensus Document on Management of Peripheral Arterial Disease (TASC) advocates endovascular treatment for TASC A and B lesions in femoral artery [4]. Moreover, unlike the TASC I Consensus, the TASC II update allows for more flexibility in TASC C lesions with respect to primary treatment with endovascular therapy based on patient factors. As for TASC D lesions, vein bypass remains to be the standard option for TASC D lesions, but it may be inappropriate for patients with severe medical comorbidities or lack of suitable vein conduits [5, 6].

With continuing advances in techniques and facilities, endovascular treatment enabled complex, long-segment occlusions to be revascularized successfully. Some reports showed good technical success rate and low perioperative complications in long SFA (superficial femoral artery) lesions [7, 8]. But, the follow-up time of these studies was short. Moreover, until now, few articles are focusing on the outcomes of primary stent-intervention on TASC-D lesions. Although primary balloon angioplasty with selective stenting is used in less severe lesions [9], primary stenting is still the most common treatment for long occlusions in the SFA. So, here we reported our experience on endovascular therapy with stenting for ASC II D femoropopliteal lesions with a long follow-up time (mean, 12.2 ± 6.1 months; range, 5 to 38 months).

## Methods

### Patients

From January 2011 to March 2013, 53 patients with 58 limbs involving TASC II D femoropopliteal lesions were retrospectively enrolled in this study. Of these limbs, 30 were lifestyle-limiting claudication (Rutherford category 2/3), 17 were rest pain (Rutheford category 4), and 11 were gangrene (Rutherford category 5). Patients who had experienced any endovascular or bypass procedures prior to this study and patients with occlusion of iliac or common femoral artery were excluded.

### Procedures

Endovascular interventions were performed under local anesthesia by our vascular surgeons. Access to the culprit lesion was achieved by way of a crossover approach using a dedicated 6 F-long sheath (45 cm) (COOK, USA). Antegrade approach and subintimal recanalization were usually used to gain access to the distal true lumen. If necessary, SAFARI techniques (subintimal arterial flossing with antegrade-retrograde intervention) were performed to improve technical success. Retrograde access was gained in the distal target artery (popliteal, anterior tibial, peroneal, or posterior tibial) and a retrograde subintimal channel was created. A hydrophilic 0.035-inch guide wire was used to connect the retrograde and antegrade subintimal channels simultaneously to create a “flossing” guide wire. Predilation with small balloon catheter was carried out. Stents were routinely deployed and lesions were treated with as few stents as possible. Adjacent stents were overlapped by 1 cm. Three types of nitinol self-expanding stents: Protégé Everflex (ev3 Inc., USA), Lifestent (Bard Inc, USA), and SMART Control (Cordis Corporation) were used according to operator discretion. Stents were routinely post-dilated to ensure optimal extension and apposition. The balloon dimension was chosen 1 mm narrower than the vessel diameter to reduce medial damage, and the balloon length should not exceed that of the stent [10].

Clopidogrel was administered 75 mg/d at least 3 days before operation, and maintained for a minimum of 6 months with 100 mg/day aspirin.

Technical success was defined as that the lesions treated had < 30 % residual stenosis at the end of the procedure. Here, the technical success is not involved in the situations of runoff vessels. Primary, assistant and secondary patency rates were determined with the SVS (Society for Vascular Surgery) reporting standards for dealing with lower extremity ischemia [11]. In accordance with this standard, primary patency rate was defined as the percentage of patients without any re-stenosis or occlusion in the arterial segment undergoing intervention during the follow-up period. Assisted-primary patency rate was defined as the percentage of patients without re-stenosis or occlusion and patients who achieved patency via additional endovascular interventions in the arterial segments suffering re-stenosis. Secondary patency rate was defined as the percentage of patients without re-stenosis or occlusion and patients achieved patency utilizing additional endovascular interventions in the occluded arterial segments. Re-stenosis was defined as > 50 % luminal diameter loss seen on angiography or duplex scanning, or 4 times ratio of the proximal normal segment velocity.

### Follow-up

Ankle brachial index (ABI), clinical examination and ultrasound imaging were evaluated initially within 48 h after surgery and repeated at 1, 3, 6, 9 and 12 months, followed by testing yearly.

### Statistical analysis

All data were analyzed using the Statistical Package for the Social Sciences (SPSS) software (SPSS Inc., Chicago, IL, USA). Continuous variables were presented as mean ± SD while categorical variables as count and percent. Demographic and co-morbidity data were recorded for every patient and patency data were calculated on a per limb basis. Primary patency, assisted-primary patency and secondary patency rates were analyzed using Kaplan-Meier analysis with Log-rank test. Univariate analysis with a cox proportional hazards model was performed to identify risk factors for re-stenosis/occlusion. A *p*-value < 0.05 was considered statistically significant.

## Results

### Characteristics of the patients and limb lesions

From January 2011 to March 2013, a total of 58 limbs in 53 patients underwent endovascular treatment. The mean age of the enrolled patients was 74.2 ± 8.2 year (range, 58.0–91.0 years). Most patients were males (67.9 %). Among the 53 patients, 32 (60.4 %) had diabetes mellitus and 36 (67.9 %) had high blood pressure (Detailed in Table 1). Mean lesion length were 314.8 ± 64.3 mm and all lesions were totally occluded before treatment. Of the 58 limbs, 30 limbs were presented with intermittent claudication, 17 were rest pain, and 11 were ulcer or minor gangrene. A total of 145 stents were implanted in SFA with an average of 2.5 stents per limb. Twenty-five limbs had only one-vessel runoff below the knee, 21 limbs had two-vessel runoff, 10 limbs had three-vessel runoff and 2 limbs had no-vessel runoff (Detailed in Table 1).

**Table 1:** Characteristics of the patients and lesion

Characteristics	Value
Patients	
Number of patients (limbs)	53 (58)
Age (years, mean ± SD)	74.2 ± 8.2
Male gender (%)	36 (67.9)
Diabetes mellitus (%)	32 (60.4)
Hypertension (%)	36 (67.9)
Coronary heart disease (%)	15 (28.3)
Hyperlipidemia (%)	19 (35.8)
Others (cerebral infarction) (%)	7 (13.2)
Lesion (all 58 limbs)	
Length of lesion (mm, mean ± SD)	314.8 ± 64.3
Average number of stents per limb	2.5
Run-off vessels below the knee (0/1/2/3)	2/25/21/10
Preoperative symptoms (intermittent claudication/rest pain/gangrene)	30/17/11
Follow-up time (months, mean ± SD)	12.2 ± 6.1

### Immediate outcomes after intervention

The over-the-bifurcation (crossover) approach was applied for 48 limbs (82.8 %) and retrograde approach (SAFARI techniques were utilized through ipsilateral popliteal artery) for 10 limbs (17.2 %). No major complications like death, cardinal infarction, acute renal failure or amputation were occurred during operations. But there were 6 (11.1 %) minor complications, including two haematomas at the puncture site (3.7 %), one acute renal insufficiency (1.9 %), one contrast media induced-encephalopathy (1.9 %), and 2 anginas (3.7 %). Fortunately, all these minor complications were cured, and no further treatment was needed.

### Outcomes during follow-up

All patients were followed up with the mean time of 12.2 ± 6.1 months (range from 5 to 38 months). There were 2 patients died during the follow-up, one from cardiac infarction at the 7th month, and the other from cancer at the 13th month. The baseline ABI was 0.49 ± 0.1 and increased to 0.93 ± 0.2 at 1 month after intervention and remained at 0.89 ± 0.2 at 1 year follow-up. All lifestyle-limiting claudication symptoms transited into mild to moderate claudication. The symptoms of rest pain occurred in 16 limbs also transited into mild to moderate claudication but one limb remained to present with rest pain. The symptoms of gangrene transited into mild to moderate claudication in 10 limbs and into rest pain in 1 limb.

**Fig. 1:**
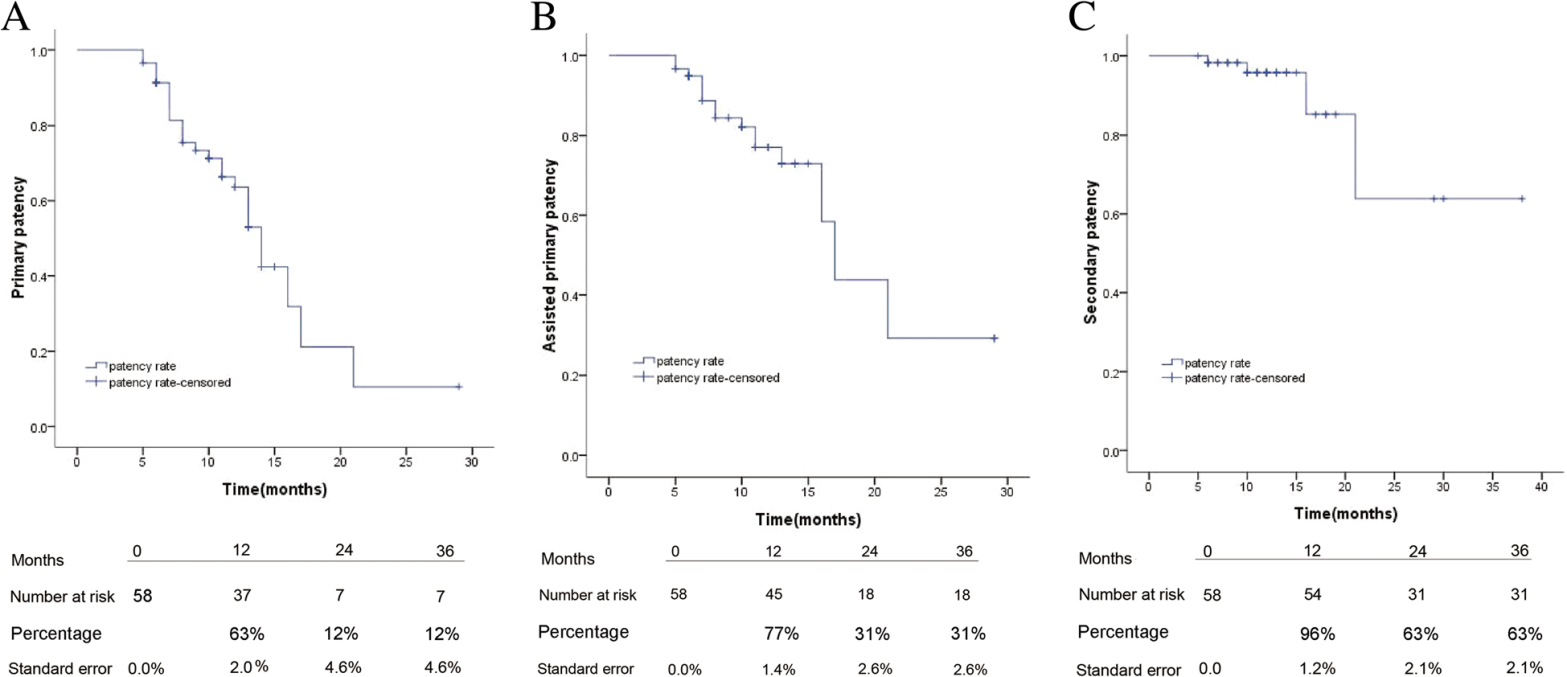
Kaplan-Meier analysis of the patency rate following endovascular intervention. **a** primary patency, **b** assisted primary patency, **c** second patency

During the follow-up period, 8 limbs experienced re-stenosis and 4 occlusions. Ten (7 re-stenosis and 3 occlusions) occurred within 1–2 years and 2 occurred (1 re-stenosis and 1 occlusions) within 2–3 years. The-primary patency rates at 1, 2 and 3 years were 63 %, 12 % and 12 %, respectively (Fig. 1a). Reintervention was performed for all limbs suffering from in-stent-re-stenosis (ISR) during the follow-up period and the vessels were successfully revascularized but 2 limbs in 2 patients. Of these 2 patients who suffered a slight rest pain, one was technically failed and received an oral drug medication since this patient had no vessel runoff that surgery bypass was unsuitable. Palliative treatment was carried out for the other patients due to severe and life-threatening comorbidities. Four patients received at least 2 reinterventions during the follow-up period and until now target lesions still remain unobstructed. Assisted primary patency rates at 1, 2 and 3 years were 77 %, 31 % and 31 %, respectively (Fig. 1b). The 4 patients with occlusions underwent a CDT (catheter directed thrombolysis) re-intervention and subsequent PTA (percutaneous transluminal angiography). No further stent implantation was needed. The secondary patency rate was 93 % at 1 year, 54 % at 2 year and still 54 % at 3 year (Fig. 1c).

During the follow-up, no major amputation was occurred. Five patients (9.3 %) underwent toe dissection due to toe gangrene and achieved a quick wound healing after intervention.

### Risk factors for re-stenosis/occlusion

Univariate analysis revealed that number of run-off vessels was associated with re-stenosis/occlusion because significantly higher risk of stenosis/occlusion in patients with two run-off vessels was found compared to patients without run-off vessel (*P* = 0.046). Other factors, such as age, gender, diabetes mellitus, hypertension, hypelipidemia and lesion length had no relationship with the occurrence of re-stenosis or occlusion (Table 2).

**Table 2:** Predictive factors for stenosis/occlusion

Factors	HR	95 % CI	*P* value
Age	0.10	0.96–1.04	0.97
Gender	1.23	0.55–2.76	0.62
Diabetes mellitus	0.84	0.40–1.72	0.63
Hypertension	0.73	0.36–1.51	0.40
Coronary heart disease	1.25	0.59–2.69	0.56
Hyperlipidemia	0.73	0.33–1.65	0.46
Cerebral infarction	1.14	0.44–2.98	0.79
Lesion length	1.00	0.10–1.01	0.82
Number of stents	0.96	0.58–1.58	0.96
Number of Run-off vessel (reference: 0 run-off vessel)			0.24
1 run-off vessel	0.23	0.05–1.06	0.06
2 run-off vessels	0.20	0.04–0.97	< 0.05
3 run-off vessels	0.31	0.06–1.61	0.16
Intermittent claudication	1.23	0.60–2.53	0.57
Rest pain	1.06	0.48–2.34	0.88
Gangrene	0.84	0.32–2.22	0.84

Note: only number of run-off vessels was detected as potentially predictive factor, patients with two run-off vessel had significantly higher risk of stenosis/occlusion compared to patients without run-off vessel (*P* = 0.046)

## Discussion

In this retrospective study, all enrolled patients were successfully revascularized and followed. In fact, there were still 3 patients who were not recruited in this study because of failure of intervention. So, the real technical success rate was 95 %. When considering that the primary goal of this study was to determine the patency rate and durability of successful endovascular interventions on TASC II D lesions, we excluded the patients who experienced unsuccessful primary interventions from this analysis.

The TASC II consensus reported in 2007 recommends surgical treatment over endovascular treatment in the case of TASC D lesions [4]. But in recent years, continuous advances in endovascular techniques and facilities have made the treatment paradigm shifted. Endovascular techniques have been gradually used for more extensive femoropopliteal occlusive disease and gained favorable outcomes [12, 13]. Baril *et al*. reviewed the data of 79 limbs from 74 patients with TASC II D lesions treated with subintimal angioplasty and stents. In their study, the primary, assisted primary and secondary patency rates at 12 months were 52.2 %, 88.4 %, 92.6 %, respectively. Our study displayed a comparable outcome that the primary, assisted primary and secondary patency rates at 12 months in current study were 63 %, 77 % and 96 %, respectively. However, the satisfactory patency rate of endovascular therapy for comprehensive lesions was tempered by significant rates of re-stenosis and occlusion. At the 24th month, the primary patency rate was dramatically decreased to 12 % in current study, lower than that (27.5 %) reported by Baril *et al*. [12]. This implied that nearly 9/10 patients developed re-stenosis or occlusion within 2 years after intervention. Fortunately, all these lesions can be treated with endovascular techniques and be more easily crossed along the stent path to the distal true lumen compared to debut performance. Furthermore, with close surveillance, early re-stenosis can be easily found and re-treatment can be performed as soon as possible that a large number of the limbs suffering re-stenosis or occlusion can be salvaged. Thus, the secondary patency rate at 12 months reached up to 96 % and 63 % at 2 years in current study. Yin *et al*. [13] showed more satisfactory outcomes that the secondary patency rate was 95 % at 2 years and maintained at 83 % at 3–4 years after intervention. In contrast, our result was somewhat discouraging. The insufficient surveillance time may be one reason when considering that the surveillance time of majority patients was short, around 12 months. Though the secondary patency rate at 2 years was not so satisfactory, no major complications occurred during the operation and no major amputations occurred during the follow-up time in our study. The limb salvage was excellent. Therefore, we consider that endovascular treatment may be the first choice treatment even in femoropopliteal TASC II D lesions.

Besides, the preferential use of primary stenting vs selective stenting during endovascular treatment for TASC D lesions is still controversial. Some authors considered that there was no additional benefit from primary stenting while many studies have advocated the preferential use of primary stenting for longer lesions (TASC C and D lesions). Surowiec et al. have shown that there was no difference in outcomes (patency or limb salvage) between patients in whom primary stenting was used compared to those in whom selective stenting was used [9]. However, a previous meta-analysis compared the outcomes of balloon angioplasty with optional stenting vs. routine primary stenting for femoropopliteal occlusive disease and suggested that primary stenting can be used as a first-line endovascular treatment for symptomatic disease in the femoropopliteal segment, mainly for long lesions [14]. Due to the differences in patients, producers, analytical method among different studies, few studies can be used to compare. Therefore, randomized clinical trials are needed to examine whether primary stenting is superior to selective stenting for type D lesions treatment.

The Outback catheter is a kind of catheter system designed to allow fluoroscopically controlled re-entry of the true arterial lumen after subintimal guidewire passage during recanalization procedures, which was safe and effective in complicated recanalization procedures [15–17]. But in our hospital, this device is not widely used due to high cost. What we can utilize is retrograde penetration and SAFARI technique. In our center, SAFARI technique was carried out for 10 limbs and all lesions were successfully crossed, leaving no major complications postoperatively. Hua *et al*. reported SAFARI-assisted stenting for 38 cases of long chronic total occlusion (CTO) of TASC C and D superficial femoral lesions and no major complications occurred as well [18]. The SAFARI technique is a safe and feasible option for patients with infrainguinal CTO, especially TASC II D lesions.

Univariate analysis showed that number of run-off vessels was associated with re-stenosis/occlusion, which was in line with the previous study [19]. However, further studies were needed for exploring whether number of run-off vessels is independent risk factors for re-stenosis/occlusion in patients with TASC II D femoropopliteal occlusive disease because multivariable analysis was inappropriate to perform with only 12 endpoints in this study. It should be noted that cerebrovascular disease and hypercholesterolemia have been evidenced to be independent predictors for re-stenosis or occlusion [12]. Besides, lesion length was also showed to be involved in the occurrence of re-stenosis or occlusion in a recent study [13]. But in current study these conclusions were not confirmed. The reasons for the difference results are unclear and further studies are needed to resolve this issue. In addition, a previous study [20] has demonstrated that there is a considerable risk of stent fractures after long segment femoral artery stenting. So, in current study we also investigated the occurrence of stent fractures considering that the objects of our study were very long SFA lesions. Stent fractures were occurred in 5 cases and there was no displacement and transaction. It seemed that there was no obvious relationship between stent fracture and restenosis since only 1 of these 5 cases showed restenosis.

This study has a number of limitations. The most important one is the limited power of the analysis due to its retrospective nature. Moreover, this was not designed as an intention-to-treat study and the primary goal of this article was to determine whether primary intervention could be the first choice rather than a backup on TASC II D lesions. Thus, patients who did not undergo successful primary interventions were excluded from the analysis. In addition, with the limitation of only 12 endpoints, multivariable analysis was inappropriate to perform for exploring independent risk factors in this study. Furthermore, the follow-up time is still limited, and longer-term follow-up will be necessary to determine the durability of these interventions.

## Conclusion

Endovascular treatment for TASC II D lesions is safe and can be effectively performed with acceptable hemodynamic improvement. Though re-stenosis is very common, close surveillance and repeated intervention can resolve this problem.

### Highlights

1. Endovascular intervention with bare nitinol stent was performed for TASC II D lesions.
2. The secondary patency rates reached up to 96 % at 1 year after intervention and maintained at 63 % to 3 years.
3. One-runoff vessels may an independent predictor for re-stenosis/occlusion.
